# Correctional Health and Oncologist Perspectives on Strategies to Improve Cancer Care in US Prisons: A Qualitative Study

**DOI:** 10.1001/jamanetworkopen.2025.37640

**Published:** 2025-10-15

**Authors:** Christopher R. Manz, Brett Nava-Coulter, Emma Voligny, Daniel A. Gundersen, Alexi A. Wright

**Affiliations:** 1 Department of Medical Oncology, Dana-Farber Cancer Institute, Boston, Massachusetts; 2 Harvard Medical School, Boston, Massachusetts; 3 Rutgers Institute for Nicotine and Tobacco Studies, New Brunswick, New Jersey; 4 Department of General Internal Medicine, Rutgers Robert Wood Johnson Medical School, New Brunswick, New Jersey

## Abstract

**Importance:**

Individuals diagnosed with cancer while incarcerated in US prisons have worse mortality than nonincarcerated individuals. Incarceration poses numerous barriers to care, and optimal strategies to improve cancer screening, diagnosis, and treatment are needed.

**Objective:**

To investigate perspectives among correctional health and oncology clinicians on strategies to improve cancer care in US prisons, from screening and diagnosis to treatment and end-of-life care.

**Design, Setting, and Participants:**

This qualitative study conducted interviews across 16 US state and federal prisons systems between September 2023 and April 2024 and a member-checking focus group in February 2025. Semistructured interviews were conducted with prison medical directors, primary care clinicians, and oncologists involved in delivering cancer care for individuals incarcerated in US prisons. Participants were recruited via email invitation sent to attendees of a correctional health conference, purposeful sampling using targeted emails to clinicians in diverse prison systems, and snowball sampling of individuals from research and participant networks. Interview transcripts were analyzed to identify all recommended strategies for improving care across the cancer care continuum. Data were analyzed from September 2023 to February 2025.

**Main Outcomes and Measures:**

To identify recommendations on tools, practices, or policies to improve cancer care (hereafter, *strategies*) for incarcerated patients with cancer.

**Results:**

A total of 32 participants from 16 prison systems were interviewed (median [range] age, 49 [33 to ≥70] years; 20 female [62.5%]; 6 Asian or Pacific Islander [18.8%], 5 Black [15.6%], and 21 White [65.6%]), including 9 medical directors, 6 primary care physicians, and 15 oncologists. Participants identified strategies to improve care addressing nearly all previously identified barrier themes, including strategies for (1) conducting screening in prisons; (2) bringing cancer treatment into prisons and centralizing care; (3) using telehealth, navigators, and early oncologist involvement to improve care coordination; (4) improving communication and social support; (5) improving symptom management, access to palliative medicine, and end-of-life care; and (6) delivering patient-centered care. Strategies require implementation by prisons, prison clinicians, oncologists, oncology practices, and policymakers. A total of 22 focus group participants reinforced these strategies with additional supportive insights.

**Conclusions and Relevance:**

In this qualitative study of clinicians delivering cancer care for incarcerated individuals, participants identified pragmatic strategies to improve cancer screening and treatment that address most identified barriers to care in US prisons. Application of these strategies may be associated with mitigation of disparities in cancer survival for incarcerated individuals.

## Key Points

**Question:**

What ways to improve cancer screening, diagnosis, and treatment in US prisons do correctional health and oncology clinicians recommend?

**Findings:**

In this qualitative study with 54 participants, prison medical directors and clinicians identified pragmatic strategies to improve cancer care delivered in prisons through screening, centralized and prison-based cancer treatment, and improved care coordination, communication, and symptom management, as well as practices and policies to promote patient-centered care.

**Meaning:**

This study found participant-identified strategies that may be associated with mitigation of considerable disparities in cancer survival for incarcerated individuals.

## Teaser

This qualitative study investigates correctional health and oncology clinician recommendations for improving cancer care in US prisons.

## Introduction

Cancer is the leading cause of death in US prisons, and individuals who are incarcerated when diagnosed with cancer have worse survival compared with individuals who have never been incarcerated at diagnosis.^1,2^ A high-quality study of Connecticut’s unified prison and jail system found that individuals who were incarcerated at cancer diagnosis had 92% higher mortality than individuals without an incarceration history.^3^ This survival disparity was markedly higher than what was observed in English and Welsh prisons; that prison system’s cancer care was critically evaluated in studies published in 2024,^4,5,6^ and individuals incarcerated at diagnosis had 16% higher mortality than nonincarcerated individuals. These qualitatively similar yet quantitatively contrasting findings suggest that incarceration itself may be associated with worse cancer outcomes and that differences in health care access prior to incarceration, carceral policies, and correctional health care may attenuate incarceration-related cancer disparities. Unfortunately, outside of English and Welsh prisons, few studies have evaluated how cancer care is delivered in prison.

In the companion study to this study,^7^ interviews with prison medical directors, clinicians, and oncologists from 16 US prison systems identified numerous barriers to care, including challenges with care coordination, communication, symptom management, transportation, understaffing, and nontransparent care within prisons. These barriers echoed findings from the only comparable study of barriers to cancer care in prisons that we identified, which evaluated English and Welsh prisons.^5^ That research team subsequently identified 5 strategies to improve cancer care focused on screening, diagnostic pathways, care coordination and continuity, and improving the treatment experience.^6^ However, given the many differences between US vs English and Welsh health care and carceral policies, research on strategies specific to US prisons is necessary. In this study, we interviewed prison medical directors and primary care clinicians (PCPs) and oncologists involved in treating incarcerated patients with cancer to identify strategies to improve cancer care in prison across the cancer continuum, from screening to end-of-life care.

## Methods

### Study Overview

In this qualitative study, we interviewed clinicians involved in cancer screening and treatment for individuals incarcerated in US prisons and conducted a focus group to determine participant perspectives on how cancer care is delivered, barriers and facilitators to cancer care, and strategies to improve care. This study summarizes results regarding strategies to improve cancer care in US prisons. This study was approved by the Dana-Farber/Harvard Cancer Center Institutional Review Board and was conducted in accordance with Standards for Reporting Qualitative Research (SRQR) reporting guideline. Participants provided verbal informed consent.

The study population, recruitment strategy and interview guide are described in detail in the companion study^7^ and in the eMethods in Supplement 1. In brief, we recruited prison medical directors, PCPs, gynecologists, palliative care clinicians, and medical, radiation, and gynecologic oncologists involved in providing cancer care to individuals incarcerated in US prisons. Participants were recruited via an email sent to attendees of a national correctional health care meeting, purposeful sampling using targeted emails to clinicians in diverse state and federal prison systems, and snowball sampling of individuals from research and participant networks. Recruitment and interviews occurred between August 2023 and April 2024. After we obtained informed consent, interviews were conducted in person, over the phone, or through videoconference. The semistructured interview guide asked participants about self-identified demographics (including gender and race), how cancer care was delivered in prison, and barriers and facilitators to care delivery, which often prompted responses about strategies to improve care (eAppendix 1 in Supplement 1). Participants described their race, and options were Asian or Pacific Islander; Black, African, or African American; and White. Race was assessed given that Black individuals are disproportionately incarcerated in US prisons and participant race could influence their perspective on study topics. We also asked participants about strategies to improve cancer care in prisons, then whether common tools used to improve care in community practices (eg, patient navigators) would be helpful if applied to the prison population. Participants were offered $200 as remuneration. Recruitment was stopped based on thematic saturation for barriers and facilitators of cancer care, which occurred after a substantial decrease in identification of new strategies to improve care.^7^

### Focus Group

We recruited a focus group for a member check to assess the credibility of study findings.^9^ We sent an email invitation to individuals who participate in a monthly correctional health videoconference for prison medical directors and clinicians. In February 2025, we conducted a 60-minute focus group. One investigator (C.R.M.) presented study results and prompted discussion after each results section presented (ie, logistics, barriers and facilitators to care, and strategies to improve care) (eAppendix 2 in Supplement 1). Two investigators (C.R.M. and A.A.W.) took notes, which were summarized to identify new strategies to improve care.

### Statistical Analysis

Audio recordings were transcribed and uploaded to NVivo software version 14 (QSR International). One investigator (B.N.C.) coded all examples or recommendations that participants mentioned of tools, practices, policies, or other recommendations to improve cancer care delivery (hereafter, collectively referred to as *strategies*), and a second investigator (C.R.M.) reviewed codes, read transcripts, and coded additional examples. Given that the objective was to identify strategies that may be useful across a wide range of correctional health contexts, we did not analyze data to identify themes and instead included all strategies, irrespective of frequency. An oncologist investigator (C.R.M.) then mapped strategies to components of cancer care delivery addressed (eg, screening) and stakeholders (prisons or correctional health clinicians, oncology practices or clinicians, and correctional health policymakers) required to enact the strategy, which were reviewed by a second oncologist investigator (A.A.W.); differences were resolved by discussion until consensus was reached. Similarly, investigators identified whether any strategies addressed each barrier at each level of influence (individual, interpersonal, institutional, and policy levels).^7,8^ Researcher characteristics are described in the eMethods in Supplement 1.

## Results

We enrolled 32 participants (median [range] age, 49 [33 to ≥70] years; 20 female [62.5%]; 6 Asian or Pacific Islander [18.8%], 5 Black [15.6%], and 21 White [65.6%]), including 9 prison medical directors (28.1%), 15 oncologists (7 radiation oncologists [21.9%], 6 medical oncologists [18.8%], and 2 gynecologic oncologists [6.3%]), and 6 primary care clinicians (18.8%) (Table 1). Participants represented 16 state and federal prison systems.

**Table 1.** Participant Characteristics

Characteristic	Participants, No. (%) (N = 32)
Age, median (range), y	49 (33 to ≥70)
Gender	
Female	20 (62.5)
Male	12 (37.5)
Race	
Asian or Pacific Islander	6 (18.8)
Black, African, or African American	5 (15.6)
White	21 (65.6)
Clinical role	
Prison medical director	9 (28.1)
Prison primary care physician	6 (18.8)
Radiation oncologist	7 (21.9)
Medical oncologist	6 (18.8)
Gynecologic oncologist	2 (6.3)
Palliative care clinician	1 (3.1)
Gynecologist	1 (3.1)
Prison systems, No.	16
Participants per system	
Texas	5 (14.7)
Indiana	4 (11.7)
Massachusetts	4 (11.7)
North Carolina	3 (8.8)
Rhode Island	3 (8.8)
California	2 (5.8)
Tennessee	2 (5.8)
Federal Bureau of Prisons	1 (2.9)
Florida	1 (2.9)
Idaho	1 (2.9)
Kansas	1 (2.9)
New Jersey	1 (2.9)
Oregon	1 (2.9)
South Dakota	1 (2.9)
Wisconsin	1 (2.9)
Wyoming	1 (2.9)

Participants suggested many strategies to improve care throughout the cancer continuum (Table 2; eFigure in Supplement 1); illustrative quotes are in the eTable in Supplement 1. Collectively, these strategies addressed most barriers described in the companion study^7^ at most levels of influence, with the exception of carceral settings’ prioritization of security over health (Figure).

**Table 2.** Suggested Strategies to Improve Care

Strategies	Stakeholder		
	Prisons and correctional health clinicians	Oncology practices and oncologists	Correctional health policymakers (government and nongovernment)
Screening	Increase prison-based testing Build EHR screening templates Plan screening drives Educate patients and clinicians	NA	Create standards and enforceable metrics for cancer screening in prison Adopt or expand USPSTF guidelines for prisons
Cancer treatment	Bring cancer care into the prison Facilitate decarceration	Bring cancer care into the prison Centralize care within oncology practices Increase clinical trial access Emphasize patient education Create oncology 101 guide for prisons	Bring cancer care into the prison Facilitate decarceration Increase clinical trial access Create standards and enforceable metrics for cancer treatment in prison Require third-party quality monitors Reduce medication costs
Care coordination	Use telehealth Involve oncology expertise early in care Invite oncologists into prison Establish navigation for patients and prisons Streamline administrative processes for care approval Use care trackers for patients with cancer Prioritize cancer care Encourage proactive clinicians Tailor reentry procedures	Use telehealth Get involved in care workup and diagnosis Conduct in-prison patient visits Establish navigation for patients and prisons Provide EHR access to prisons for more timely communication of clinic notes	Streamline administrative processes for care approval
Communication	Establish direct communication between prison clinicians and oncologists Create peer support groups and train peer educators Allow greater family involvement	Establish direct communication between prison clinicians and oncologists Emphasize patient education	Allow greater family involvement
Symptom management, palliative care, and end-of-life care	Increase access to palliative care and hospice Facilitate decarceration and medical parole near the end of life Increase medication access Create peer support groups and train peer educators	NA	Increase access to palliative care and hospice Facilitate decarceration and medical parole near the end of life
Patient-centered care	Allow greater family involvement Liberalize physical-restraint policies Take a trauma-informed approach to cancer screening and treatment	NA	Allow greater family involvement Liberalize physical-restraint policies

Abbreviations: EHR, electronic health record; NA, not applicable; USPSTF, US Preventive Services Task Force.

**Figure.**
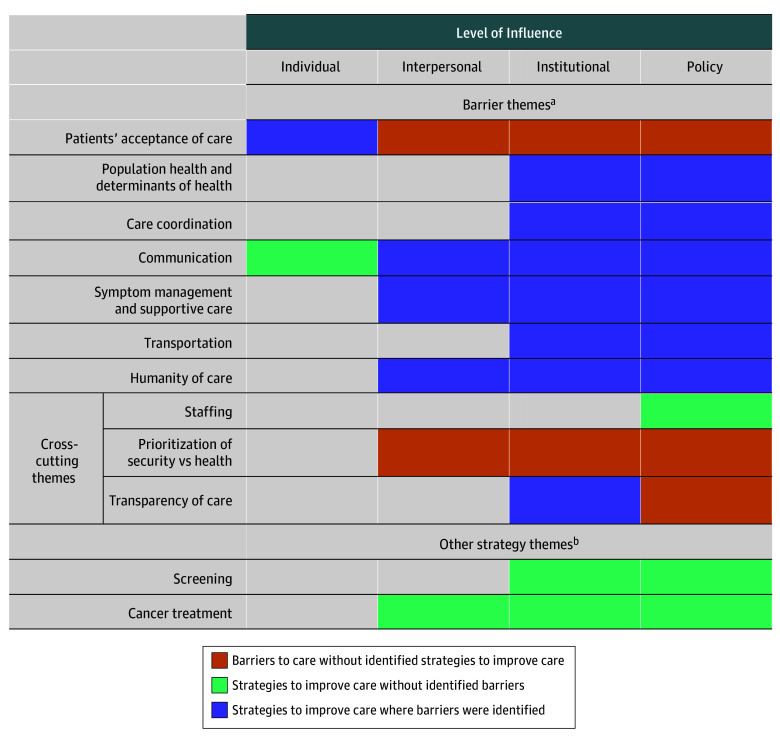
Suggested Strategies to Improve Prison Cancer Care Mapped on Barriers to Care Themes related to barriers to care are listed on the left, with colored rectangles indicating the level of influence at which a barrier acts and whether a strategy may be associated with improved care at that level of influence. Levels of influence are based on a conceptual framework for health disparities.^8^ ^a^See Manz et al^7^ for source of barrier data. ^b^Strategies that do not address 1 specific barrier theme but instead apply to screening or cancer care generally.

### Cancer Screening

#### Screening Prompts and Education

Participants indicated that prison electronic health records (EHRs) should incorporate reminders to prompt screening. Participants also encouraged educational outreach to clinicians focused on cancers that are common among incarcerated individuals and symptoms and clinical findings that should prompt cancer evaluations, paired with tailored education materials for incarcerated individuals.

#### Screening Within Prisons

Participants stated that administering screening tests inside prisons increases adherence. For instance, medical director I reported, “a lot of people don’t want to do [mammography outside of prison] because they’re embarrassed by [being shackled and wearing orange jumpsuits in public]… So as much as we can bring in the mammography unit [into the prison, as we did] more than 10 years ago, it just increased the compliance of patients enormously.” Participants reported that portable imaging could also improve screening rates by eliminating care coordination and transportation barriers. For tests that must occur outside of prisons (eg, colonoscopies), preplanned screening drives enable coordination with security and transportation teams and avoids disruptive, unplanned resource use, participants said.

#### Screening Standards

Participants emphasized a need for clear, enforceable standards for cancer screening in prisons that can be incorporated into contracts with correctional health organizations. They also highlighted the importance of applying US Preventive Services Task Force guidelines, as well as liver cancer screening, because incarcerated individuals have higher rates of liver cancer than never-incarcerated individuals.

### Cancer Treatment

#### Prison-Based Care

Participants emphasized that bringing cancer care into prisons, although challenging, would reduce many barriers to treatment. Participants suggested that oncologists may build rapport with patients and improve their understanding of their patients’ experiences by occasionally seeing them in prison. Gynecologic oncologist B noted, “There was something about making the trip out to the prison and seeing patients in their own space that … added to the therapeutic relationship and allowed me to really understand why something was a barrier.” Participants also suggested that administration of systemic therapies in prison would eliminate transportation barriers while increasing adherence to treatment.

#### Care Outside of Prison

Outside of prison, participants recommended centralizing care among teams experienced in caring for incarcerated patients. Several participants reported that oncologists could address communication gaps with patients by providing clear and thorough education about patient diagnoses, prognoses, and treatment goals.

#### Policies

Participants suggested that policymakers should improve care by creating enforceable standards for cancer treatment in correctional settings. One participant referenced the American College of Surgeon Commission on Cancer standards as an analogy. Others recommended that policymakers enable prison-based cancer treatment, reduce costs of cancer medicines, and eliminate legislative barriers to clinical trials. Several others highlighted the value of having third-party quality monitors involved to ensure accountability. Medical director F described using quality monitors to advocate for patients when prisons face difficulty arranging timely care in the community. Participants pointedly stated that prisons are not set up to deliver high-quality cancer care and that patients and prisons alike may be better served by decarcerating patients with cancer who no longer pose safety risks.

### Care Coordination

#### Early Oncologist Involvement and Telehealth

Many participants noted that oncology input on diagnostic testing prior to oncology visits was helpful. Several participants reported that telehealth facilitates oncology involvement throughout the care continuum while eliminating the need for transportation and security. Telehealth can be coupled with physical exams by in-prison clinicians and prison-based lab testing to extend opportunities for telehealth, participants said.

#### Navigation and Care Tracking

Several participants suggested that prisons should employ navigators to assist with ensuring timely care and coordination with cancer practices. They also recommended using dashboards or registries to track patient care plans and ensure adherence to treatment and surveillance schedules. Several participants recommended that prison clinicians should also proactively engage oncology staff regarding care plans. However, gynecologist A cautioned that relying on individual clinicians to be proactive instead of creating systemic solutions contributes to burnout.

#### Scheduling Processes

Participants recommended that prisons and policymakers further facilitate care by streamlining approval processes for diagnostic workups (eg, bundled approvals for all tests and oncology visits needed for diagnosis). They also reported a need for modernized prison EHRs that integrate scheduling and ensure that annual surveillance and screening tests do not fall through the cracks. Given transportation limitations, many participants reported that their prisons prioritized patients with time-sensitive cancer treatment plans over individuals with chronic conditions. Additionally, several participants raised the importance of assisting patient transition to community-based care after prison release, guided by input from the treating oncologist and addressing preincarceration barriers to care that may reemerge upon release (eg, transportation barriers).

### Communication

#### Direct Communication

In addition to the care coordination tools described previously, participants reported that prisons and oncology practices should establish direct communication between prison and oncology clinicians to enable clear coordination of care and better symptom management. Several participants highlighted that simply sharing oncologist cell phone numbers with prison PCPs greatly improved care.

#### Improving Social Support

Participants noted that prisons and policymakers can improve patient understanding of their cancer care, increase social support, and reduce care declination by allowing family members to participate in cancer care discussions in person or via telephone. Radiation oncologist F remarked, “Allowing [patients] the opportunity to have 1 person … to join the consultation and hear it, they deserve that because it’s a lot of information. Not everyone has the same education level, and you’re throwing out words at them that they’ve never heard before that are oncology terms.” Using telehealth for these discussions may alleviate escape concerns, which are frequently used to justify restrictions on family participation, participants said. Participants shared that prisons may also create support groups or facilitate peer education among other incarcerated individuals with cancer.

### Symptom Management, Palliative Care, and End-of-Life Care

#### Access to Symptom Medications

Noting that improvements in care coordination and communication will also facilitate better symptom management, several participants reported that prisons should improve symptom management by expanding formularies to include common cancer medicines (eg, antiemetics) and liberalizing dispensing times and types of medications eligible to be on person in a cell for individuals with cancer. Meanwhile, oncology clinicians can schedule medications instead of prescribing them as needed to ensure that patients have an opportunity to take or decline medications at scheduled dispensing times, participants suggested. Some participants reported that having patients stay in infirmaries may also improve symptom management but that relocation to the infirmary may incur social isolation and loss of privileges that may disincentivize individuals to engage in care.

#### Palliative Care and End-of-Life Policies

Participants recommended that prisons and policymakers proactively increase access to palliative care specialists and have dedicated hospice beds staffed by personnel trained in end-of-life symptom management. However, several participants reported that patients dying from cancer likely represent a low risk of harm to society and argued that prisons and policymakers should facilitate decarceration through compassionate release. For example, gynecologist A, said, “We should be reevaluating [sentencing] when someone gets a cancer diagnosis … I tend to really think that the best thing we can probably do is work on getting people out and home.” Critically, prison policies must also allow incarcerated patients the opportunity to choose do-not-resuscitate orders and otherwise opt against aggressive care, participants said.

### Patient-Centered Care

Participants noted that incarcerated individuals have often been exposed to numerous traumas and that a trauma-informed approach to health care may engender greater trust, patient understanding, and buy-in from patients with their care. This approach includes reconsidering policies that expose women to default pelvic exams irrespective of recent cervical cancer screening (eg, upon reincarceration) and facilitating family involvement. Prisons and policymakers can further promote patient-centered care by liberalizing policies on physical restraints, particularly in clinical situations where they are likely unnecessary (eg, while patients are actively dying), participants said. To reduce conscious or unconscious clinician bias based on patient criminal histories, cancer practices should also discourage clinicians from looking up these histories, participants recommended.

### Focus Group Member-Checking

The focus group consisted of 22 prison medical directors and clinicians; demographic characteristics were not obtained. Focus group recommendations supported or echoed strategies that were presented. Participants added that company policies that emphasize cancer screening and peer education can improve adherence to guidelines. They recommended implementing EHRs in prisons without such records, noted that case managers could act as navigators, and agreed that telehealth can help overcome transportation barriers, although some prisons may have low internet bandwidth or EHRs that cannot accommodate telehealth. Others noted that mechanisms for ensuring that prisons receive outside oncology notes are needed, including access to external EHRs. Participants also noted that an oncology 101 reference guide would be helpful for prison PCPs to stay abreast of cancer diagnostic evaluations, basic treatment strategies, and identification of oncologic emergencies.

## Discussion

In this qualitative study of prison medical directors, PCPs, and oncologists involved in delivering cancer care for individuals incarcerated in US prisons, participants described many strategies to improve cancer care. They highlighted the importance of implementing enforceable standards for cancer screening and treatment in prison, creating mechanisms that overcome care coordination and communication barriers between prison and oncology settings, liberalizing policies to enable better symptom management and family member involvement, and bringing cancer care into prisons, when feasible.

To our knowledge, this is one of the first studies to examine specific strategies to improve cancer care delivery across multiple US prisons. While our companion study revealed multiple barriers to cancer care in prisons across all levels of influence, this study found suggested strategies to overcome many of these barriers.^7^ For example, participants suggested that tools such as navigation, cancer care trackers, and telehealth may overcome many communication and care coordination barriers that occur in fragmented health care settings by establishing mechanisms to proactively ensure that patients receive timely care. Notably, strategies we identified include the top strategies that expert stakeholders identified to improve cancer care in English and Welsh prisons.^6,10^ Implicitly, these tools apply proven patient-navigation concepts (using deliberately designed processes and navigators to help patients and clinicians identify next steps and ensure timely treatment) to carceral settings, where patients have limited information and autonomy for self-advocacy.^11,12^ Collectively, participant recommendations amount to a call for correctional health organizations and oncology practices to implement purposive systems that, in effect, advocate for patients who have limited ability to advocate for themselves.

Several participants spoke to the need to bring humanity back into dehumanized and security-driven care systems. Participants highlighted the importance of enabling family members to participate in care and provide critical social support. Oncologists and correctional health clinicians shared that personal ties between clinicians facilitated communication and improved timely diagnosis, symptom management, and care coordination. In addition, prison- and community-based participants advocated for increased decarceration and decreased use of physical restraints, recognizing that many patients with cancer pose a low risk of harm to others. Their recommendations for increased use of compassionate release echo those of other community organizations and governmental bodies, given that data from the US Sentencing Commission and others show that compassionate release is rarely granted.^13,14,15^

Participants also spoke of the importance of trying to bring cancer care into the prison when feasible. Given that telehealth eliminates major transportation and security barriers and that in-prison clinicians can conduct physical exams, prisons and oncology practices could leverage telehealth throughout the cancer continuum, from prediagnosis through posttreatment surveillance, similar to how it has been used for other chronic disease in carceral settings.^16^ Enabling in-prison treatment administration may similarly overcome several barriers and thus may be associated with improved patient acceptance and timely administration of treatment. Having oncologists occasionally see patients within prison may be associated with improved therapeutic alliances and care given that oncologists better understand prison health care logistics.

Finally, at the policy level, participants discussed the need for enforceable care standards for cancer screening and treatment in prison. Incarcerated patients in the US have a constitutional right to health care that generally meets community standards.^17,18,19^ Arguably, many identified strategies are necessary to achieve community standards for incarcerated patients. However, establishing prison-specific care standards may require tailoring community standards to the carceral setting to account for unique aspects of the prison and differential risks for incarcerated individuals. Organizations such as the National Commission on Correctional Health Care could convene correctional health and cancer care–delivery experts and representatives for incarcerated individuals to inform such guidelines.

Participants acknowledged that multilevel interventions will be necessary to improve incarceration-related cancer disparities and that tailoring interventions to specific prison systems will require engagement from all stakeholders. Given the challenges of implementing clinical trials in correctional settings, correctional health and oncology teams can implement, evaluate, and publish quality-improvement efforts to help other prison systems learn and adapt, as in a 2025 study describing efforts to coordinate lung cancer screening caravans.^20^

### Limitations

This study has several limitations. First, the study included all participant recommendations on strategies to improve care delivery in prisons, without consideration of costs or feasibility. However, many strategies may be cost-saving due to reduced transportation and security costs (in English and Welsh prisons, security costs for out-of-prison care tripled spending for incarcerated patient cancer care, from £6589 to £20 312 annually^21^). Second, while we interviewed clinicians within 16 prison systems, there are likely additional strategies to improve cancer care that we did not identify, although our focus group identified only 2 additional strategies. Of note, the study was conducted at the level of prison systems rather than individual prisons; most participants had roles where their experiences encompassed many prisons within a prison system, and their perspectives reflect considerable variability within and between prisons in characteristics that may affect cancer care (eg, on-site care availability, security level, and rurality). Still, the feasibility of strategies may vary within and between prisons due to these characteristics. Third, our findings may not apply to jails, where cancer is a rare cause of death, populations are more transient, and health care logistics are more varied and complex.^22^ Fourth, our study did not include perspectives of incarcerated patients, whose input is an important priority for future research.

## Conclusions

In this qualitative study, clinicians involved in coordinating cancer screening, diagnosis, and treatment for individuals incarcerated in US prisons identified many strategies to improve cancer care delivery. Future studies should test optimal strategies to overcome barriers to care and reduce survival disparities associated with incarceration.

## Notes

Supplement 1.
eMethods.
**eAppendix 1.** Interview Guide **eAppendix 2.** Focus Group Guide **eTable.** Illustrative Quotes on Ways to Improve Cancer Care Delivery in Prisons **eFigure.** Alternative Summary of Strategies to Improve Care Across the Care Continuum

Supplement 2.
Data Sharing Statement
