# Supplementary material for: Correctional Health and Oncologist Perspectives on Strategies to Improve Cancer Care in US Prisons: A Qualitative Study
Source: JAMA Netw Open. 2025 Oct 15;8(10):e2537640. doi: 10.1001/jamanetworkopen.2025.37640 (PMC12529190; doi:10.1001/jamanetworkopen.2025.37640)
Supplement: Supplement 1. — eMethods. eAppendix 1. Interview Guide eAppendix 2. Focus Group Guide eTable. Illustrative Quotes on Ways to Improve Cancer Care Delivery in Prisons eFigure. Alternative Summary of Strategies to Improve Care Across the Care Continuum [file jamanetwopen-e2537640-s001.pdf]

Supplemental Online Content

Manz CR, Nava-Coulter B, Voligny E, Gundersen DA, Wright AA. Correctional health and oncologist perspectives on strategies to improve cancer care in US prisons: a qualitative study. *JAMA Netw Open*. 2025;8(10):e2537640. doi:10.1001/jamanetworkopen.2025.37640

eMethods.

eAppendix 1. Interview Guide

eAppendix 2. Focus Group Guide

eTable. Illustrative Quotes on Ways to Improve Cancer Care Delivery in Prisons

eFigure. Alternative Summary of Strategies to Improve Care Across the Care Continuum

This supplemental material has been provided by the authors to give readers additional information about their work.

© 2025 Manz CR et al. *JAMA Network Open*.

eMethods

Participant selection. Participants were recruited via three methods. First, we emailed invitations to attendees of the American College of Correctional Physicians' (ACCP) 2023 Fall Education Conference and conducted in-person interviews with interested individuals at the conference. Second, we selected several states that varied by geographic size, population, US region, and model of correctional healthcare (see next section). Through purposive sampling, we recruited participants via email from each of the seven clinical roles, identifying eligible participants through internet searches, queries to prison systems and referrals from other study participants. Third, using snowball sampling, we recruited eligible individuals via email who were identified by the research team, colleagues or study participants as likely to have unique insights into the study questions.

Selection of state prison systems: We hypothesized a priori that characteristics of state prison and correctional healthcare systems may influence the delivery of cancer care. For example:

- Geographic size: Smaller states may be able to centralize and coordinate care more easily than larger states;
- Population size: States with larger populations may have bigger prison populations with more heterogeneous cancer diagnoses, which may enable them to make more substantial investments in prison-based cancer care (e.g., on-site radiation facilities);
- Regional variation: Regions such as the Southeastern U.S. have both higher incarceration rates and worse cancer outcomes in the general population, compared with other regions, which may influence the quality of prison-based cancer care; and
- Healthcare delivery model: States that contract with for-profit companies may have different incentives for providing cancer screening and treatment than states with state-run correctional health systems.

Thus, to obtain perspectives from clinicians involved in prison systems representing these diverse characteristics, we selected five states that varied across these domains:

- Indiana: Mid-sized state and population located in the Midwest with correctional health delivered by contracted care.
- Massachusetts: Small-sized state with a mid-sized population located in the Northeast with correctional health delivered by contracted care.
- North Carolina: Mid-sized state with a large population located in the South with correctional health delivered by a mix of direct provision from the state and contracted care.
- Rhode Island: Small-sized state with a small population located in the Northeast with correctional health delivered by direct provision from the state.
- Texas: Large-sized state with a large population located in the South with correctional health delivered by direct provision from the state.

Researcher characteristics and reflexivity. Overall, none of the investigators have been incarcerated in prison or have extensive experience providing direct care to individuals incarcerated in prison. Two investigators have experience evaluating cancer care delivery and implementing interventions to improve cancer care, which may enable them to better detect when participants recommend something that could be labeled as a strategy to improve care.

© 2025 Manz CR et al. *JAMA Network Open*.

Two other investigators have experience conducting qualitative research. We used a team-based approach to study design and analysis to leverage this diversity of skills. Other researcher characteristics are unlikely to influence study findings: AAW is a non-Hispanic White medical oncologist and expert in cancer care delivery and intervention research. BNC is a bi-ethnic male sociologist with a master's degree and has multiple years of qualitative interview experience, and two years working in qualitative oncology research. CM is a non-Hispanic White male who is a medical oncologist and health services researcher. EV is non-Hispanic White and Asian female who is a research project manager. DG is non-Hispanic White immigrant and is a methodologist with multiple years of experience designing mixed methods research for cancer care delivery and related research.

© 2025 Manz CR et al. *JAMA Network Open*.

eAppendix 1. Interview Guide

Participant ID: _____________
Interview Date: ____________

Introduction:

Hi, my name is [interviewer's name]. Thank you for agreeing to participate in this interview as part of a study by Dr. Christopher Manz at the Dana-Farber Cancer Institute. We appreciate the time you are taking to share your thoughts with us.

The purpose of this study is to better understand how cancer diagnosis and treatment is delivered for incarcerated patients. Our goal is to take what we learn from you and others and condense those insights to help prison systems improve cancer care for incarcerated patients. I have a few main guiding questions, but I want to emphasize that there are no "right" or "wrong" answers. Your insights on how cancer care delivery works well and how it can be improved can be equally helpful for other prisons trying to improve care. We anticipate that up to 50 individuals will participate in this study.

Participation is voluntary. Refusal to participate will involve no penalty or loss of benefits. Reponses to this interview will not be used in another study without your permission.

This interview is expected to take 30 to 60 minutes. You will be compensated with a Visa gift card of $200 for your participation. Compensation will be sent to you via email a few days after the interview.

I would like to audio record this interview. This is simply so I can focus on our conversation and do not miss any of the feedback you provide. This conversation is anonymous and confidential; meaning that nothing you say will be linked to you as an individual and no one outside the research team will have access to the recordings. We will then transcribe and process the data to facilitate analysis. We will replace your name and location with a code. The transcription will remain anonymous, meaning we will not use your name, the name of the facility or facilities where you work, or any other information that could be used to identify you.

If you do not feel comfortable answering a question, it is ok for you to tell me that you do not want to answer. You can also choose to stop this interview at any time, and you can choose to have all records of your participation deleted.

At any time, you can contact the study lead, Dr. Christopher Manz, with any questions or concerns or about any injury related to this interview by using the information on this card. For questions about your rights as a research participant, please contact a representative of the Office for Human Research Studies at Dana-Farber Cancer Institute (617) 632-3029. This can include questions about your participation in the study, concerns about the study, a research related injury, or if you feel/felt under pressure to enroll in this research study or to continue to participate in this research study.

© 2025 Manz CR et al. *JAMA Network Open*.

Do you have any questions for me?
Do you agree to participate in this study?
I have started recording. Thank you for agreeing to participate in this study.

Demographics

1. As part of the study, we will describe the characteristics of those whom we have interviewed. These characteristics will not be stored with your responses. Will you please state your age, gender, race and ethnicity?

2. What is your role in correctional health care (e.g., medical director, primary care clinician or oncology specialist) and in what prison system do you practice (e.g., Georgia state prison or Federal Bureau of Prisons).

Topic 1: Logistics of cancer care delivery

3. We are focusing on prisons (not jails) and discussing cancer care delivery, starting with screening and diagnosis and ending with end-of-life care or survivorship. Can you please describe how cancer care is delivered for individuals incarcerated in prisonin your system along this spectrum?

Guiding questions to ensure completeness:

A. *What screening is offered and where does screening typically occur (e.g., In prison? In community facilities that are not part of cancer treatment?)? What determines who gets offered screening?*
B. *Where does diagnosis typically occur (e.g., In prison? In community facilities that are not part of cancer treatment?) and how are patients linked to cancer treatment?*
C. *Where do the following treatments primarily get delivered (e.g., in prison, at an academic hospital, at a private community clinic, etc):*
    a. *Chemotherapy*
    b. *Radiation*
    c. *Cancer surgery*
    d. *Gynecologic surgery*
D. *How is cancer treatment authorized within your system (e.g., prior authorization)? How is treatment paid for within your system (e.g., fee-for-service paid by the state? Capitated payments to certain institutions)?*
E. *How do patients access as needed symptom medications?*
F. *How is palliative care specialty care and hospice delivered, both inpatient and outpatient? Are formal palliative care and hospice services available?*
G. *Upon release from prison, are there any processes to facilitate transferring care for incarcerated patients with a cancer history to community clinicians (e.g., around insurance coverage or establishing care with a community oncologist)?*

4. I am interested in hearing about your experience overseeing, coordinating and/or treating cancer for incarcerated patients. In addition to what you have already mentioned, are there

others ways in which cancer care delivery for incarcerated patients different than providing care for similar non-incarcerated patients?

Prompt on these topics: It might be helpful to think about differences around:

- *screening*
- *diagnosis*
- *the process to initiation of cancer treatment (from the suspicion of a cancer diagnosis to actually getting treatment)*
- *treatment delivery*
- *symptom management*
- *end-of-life care and*
- *survivorship (surveillance for recurrence after completion of curative-intent treatment).*
- *the logistics of how care is delivered (e.g. transportation)*
- *how care is authorized and paid for (e.g., co-pays, prior authorization)*

If they only respond with:

A. *ways that care is worse, prompt for a response on how cancer care delivery might be better for incarcerated patients than non-incarcerated patients.*
B. *ways that care is better, prompt for a response on how cancer care delivery might be worse for incarcerated patients than non-incarcerated patients.*
C. *a description of what studies or reports say about these differences, prompt for their perception of cancer care delivery based on their experience.*

Topic 2: Barriers and facilitators to high quality cancer care

5. Please tell me about some of the things that your organization does or uses to facilitate cancer care for patients in prison.

Additional prompt: For instance, some prisons use might prioritize cancer patients for transportation, or might use patient registries to track patient care.

6. In nearly all community settings, there are barriers to care (e.g., treatment delays due to transportation difficulties) that may make cancer care more challenging. The barriers to care for incarcerated patients may be similar or different as those for patients in the community. Please tell me about barriers to cancer care for incarcerated patients under your care.

Follow-up question: Barriers to care can occur at many different levels, from the individual up to health systems and policies. What barriers come to mind at:

a. *The individual level (barriers related to the patients themselves)*
b. *The interpersonal level (barriers related to the relationship between patients and other individuals involved in cancer care, including correctional staff)*
c. *The institutional level (barriers related to the structure of prison, clinics or hospitals, e.g., staffing for transportation)*
d. *The policy level (e.g., local / state / national policies)*

© 2025 Manz CR et al. *JAMA Network Open*.

Follow-up question: We have heard from others that care coordination is a particular challenge. Can you tell me whether and how that has been the case in your patient population?

Topic 3: Utility of various care delivery mechanisms for improving cancer care for incarcerated patients

7. We have discussed many barriers to care. What tools, processes or policies might help overcome these barriers?

8. Community oncology practices often use a variety of tools to try to improve cancer care delivery, and perhaps your organization uses some of these as well. I am going to list a few common tools - if your organization uses the tool, please tell me how these might be helpful, if at all. If your organization does not use the tool, please tell me how it you think it might be helpful.
    a. Cancer care navigators (e.g., staff member dedicated to making sure patients with a cancer diagnosis receive necessary tests, appointments and treatments in a timely manner)
    b. Patient dashboards / cancer registry (e.g., a list of patients with cancer +/- ways of tracking patient care or process measures)
    c. Quality improvement processes or research focused on how to improve cancer care delivery for incarcerated patients
    d. Telehealth appointments
    e. Just prior to release from prison, a formal procedure to link cancer patients to community oncologists for continued treatment or follow-up
    f. Increased access of incarcerated patients to therapeutic clinical trials
    g. Oncologists only:
        i. Tumor boards (i.e., regular multidisciplinary meetings of medical, radiation and surgical oncologists to discuss diagnostic or treatment challenges)
        ii. Next generation genetic tumor sequencing (e.g., 100+ gene tumor mutation profiling such as Foundation One) for all patients with incurable cancer

9. Finally, some systems feel they have care delivery processes that other correctional health systems may want to emulate. Please discuss any such processes in your system.

Wrap up

10. Do you have any other thoughts that you would like to share about cancer care for incarcerated patients, especially about topics that we have not discussed that you think others would want to hear about?

11. *For medical directors:* We plan to request permission to conduct similar interviews with other individuals involved in cancer care delivery for your patients. Would you be willing

to share with us the names and organizations of an individual for each of the following specialties who might be willing to participate?

a. Primary care clinician within the correctional health organization that cares for patients in your prison system
b. Medical oncologist
c. Radiation oncologist
d. Surgical oncologist
e. Gynecologic oncologist / gynecologist

Thank you for your participation. This concludes the interview. Recording has been turned off.

12. Will you please provide your email address where we can send your $200 gift card?

© 2025 Manz CR et al. *JAMA Network Open*.

eAppendix 2. Focus Group Guide

Investigator summarizes results regarding cancer care logistics, then asks:

1. Are the findings about how cancer care is delivered generally consistent with your experiences?
2. In what ways are they different?
3. Did we miss anything that you've noticed?

Investigator summarizes results regarding barriers and facilitators to care, then asks:

4. Regarding barriers:
    a. Are the findings about barriers to cancer care for incarcerated patients generally consistent with your experience?
    b. In what ways are they different?
    c. Did we miss anything that you've noticed?
5. Regarding facilitators:
    a. Are the findings about facilitators—processes that help improve cancer care delivery for incarcerated patients—consistent with your experiences?
    b. In what ways are they different?
    c. Did we miss anything that you've noticed?

Investigator summarizes results regarding strategies to improve care, then asks:

6. Of the tools and opportunities for improving cancer care that we reviewed, which are the most promising for improving care in your care settings?
7. Are there other opportunities to improve cancer care that we have not discussed that your systems use or would considering using?

© 2025 Manz CR et al. *JAMA Network Open*.

eTable.

Table. Illustrative quotes on ways to improve cancer care delivery in prisons
**Screening**
Medical director I:<br>"One of the difficulties with something like [mammography] can be, when a prisoner goes out to an outside facility … they have to dress out in an orange jumpsuit, be shackled hand and foot with a chain in between, so a four-point shackle and have two officers… and then you go out to the outside doctor's waiting room. And a lot of people don't wanna do that because they're embarrassed by it. And that can be a problem. So as much as we can bring in the mammography unit [into the prison as we did] more than ten years ago, it just increased the compliance of the patients enormously."
**Cancer treatment**
Gynecologic oncologist B:<br>"I think there was something about me making the trip out to the prison and seeing patients in their own space that not only facilitated things, but also … added to the therapeutic relationship and allowed me to really understand why something was a barrier. Because I was seeing what it took to get from the parking lot where I parked my car to the clinic and all the layers of security and how inmates moved between various spaces. And how the infirmary looked and what the staffing of the infirmary was and what med lines looked like when patients were going for their medications. So that when patients talked about these things as problematic or not, I really had a vision in my head for what it looked like. And I think that's what – I think that is where folks who haven't worked within the system, where it can be kind of difficult to understand why things are harder in certain ways when a patient is incarcerated… But I certainly think that having a certain number of specialists actually come to the prison does facilitate care in those ways."
Medical director C:<br>"Trying to get as much care on-site as humanly possible [would help improve cancer care in prisons]. Again, because the barriers to get them to an imaging clinic or an oncology clinic, if you can get an oncologist to come into the clinic, if you can… have a hospital ward or imaging in a prison facility, that makes things a lot easier."
Radiation oncologist F:<br>"Having consequences for the prison system to face if they fail in quality measure. So a quality measure would be if someone has a diagnosis of lung cancer, they should be starting with treatment within 30 days. Not meeting that quality in a prison system should have consequences… So quality measures need to be standardized for cancer treatment delivery in prisons. And this way they can be held accountable from a prison system perspective."
Medical director C:<br>"And then I think fourth, the easiest one and best way, I think, to provide cancer care to incarcerated people that is low-hanging fruit is decarceration of individuals with cancer… And so by decarcerating and trying to set up these people that have cancers with community treatment, frankly, allows healthcare systems in prisons to be more efficient. They can kind of not have to spend so much time on these high utilizers of complex care that are cancer patients and focus on care delivery for everyone else, including cancer screening."

© 2025 Manz CR et al. *JAMA Network Open*.

Care Coordination
Medical director E:<br>"….How are you leveraging technology to track your patients? Embedding it into the electronic health record, disease management software, so …. looking at ways in which the informatics can help drive quality of care for cancer treatment would be something that I would look at [to improve care coordination]."
Medical director J:<br>"…That navigator, that would be an amazing position because we need someone with [all the medical information] on all those patients.… I don't think there are a lot of good policies in place for [making sure patients get surveillance care]. It's just so difficult."
Radiation oncologist F:<br>"The other thing is the areas in which they're failing because of staffing issues, transportation issues, logistics issues, those can be addressed with telehealth."
**Communication**
Radiation oncologist F:<br>"How we treat patients to make sure it's humane, allowing them the opportunity to have one person, even if it's on the phone to join the consultation and hear it. They deserve that because it's a lot of information. Not everyone has the same education level, and you're throwing out words at them that they've never heard before that are oncology terms. And so affording them the opportunity to have someone present is very, very important from an ethical perspective."
Medical director D:<br>"So we historically have a lot of folks who either have received very little healthcare or they had bad experiences in healthcare for one reason or another… And maybe there were bad outcomes for a family member or a friend for whatever reason. And that just kind of adds to just a lack of trust, maybe a lack of comfort with the medical community too… One way we try to get around that is, we have peer educators that sometimes can help. So if I have somebody who's in my very similar shoes, not same but similar, I might receive that a lot better from them."
**Symptom management, palliative care and end-of-life care**
Medical oncologist G:<br>"Expanding their formulary medications … really helps"
Primary care physician C:<br>"And then I just really think that we need to put more emphasis on giving them the support that they need emotionally. So whether it be from a special counselor that just maybe was special for oncology patients or if we're not going to allow them to have one designated family member to attend those appointments, or just something like that because I feel like that would just be such a lonely time for them and it's already lonely being in prison and then to get this life-altering diagnosis, just something to that affect would be nice to have that in place."
Gynecologist A:<br>"… We should be reevaluating that when someone gets a cancer diagnosis – right, like if you are in your 60s, you have whatever cancer, are you really a public safety threat? I mean, maybe, but I'd be hard pressed to find any, frankly, of those cancer patients. They may be a threat to themselves in terms of their substance use, and mental health challenges, but those

© 2025 Manz CR et al. *JAMA Network Open*.

aren't made better by being in a prison or a jail. And so I tend to really think that the best thing we can probably do is work on getting people out and home”	
**Patient-centered care**	
Radiation oncologist F:<br>“There’s no way you’ll have access to [information on criminal history] as a human being and not be biased in the way you treat the patient. And so there’s clearly some biases that need to be addressed, even from a provider perspective about how we approach these patients.”	

© 2025 Manz CR et al. *JAMA Network Open*.

Supplemental Figure

Alternative summary of strategies to improve care across the care continuum

Screening

A. Increase prison-based testing
B. Build EHR screening templates
C. Plan screening drives
D. Educate patients and clinicians
E. Create standards and enforceable metrics for cancer screening in prison
F. Adopt/expand United States Preventive Services Task Force guidelines for prison

Cancer treatment

G. Bring cancer care into the prison
H. Facilitate decarceration
I. Centralize care within oncology practices
J. Increase clinical trial access
K. Emphasize patient education
L. Create Oncology 101 guide for prisons
M. Create standards and enforceable metrics for cancer treatment in prison
N. Require third party quality monitors
O. Reduce medication costs

Care coordination

P. Use telehealth
Q. Involve oncology expertise early in care
R. Invite oncologists into prison
S. Establish navigation for patients and prison systems
T. Provide EHR access to prisons
U. Streamline administrative processes for care approval
V. Use cancer patient care trackers
W. Prioritize cancer care
X. Encourage proactive clinicians
Y. Tailor re-entry procedures

Communication

Z. Establish direct communication between prison clinicians and oncologists
AA. Create peer support groups and train peer educators
BB. Allow greater family involvement
CC. Emphasize patient education

Symptom management, palliative care and end-of-life care

DD. Increase access to palliative care and hospice
EE. Facilitate decarceration and medical parole near the end of life
FF. Increase medication access
GG. Create peer support groups and train peer educators

Patient-centered care

HH. Allow greater family involvement
II. Liberalize physical restraint policies
JJ. Take a trauma-informed approach to cancer screening and treatment

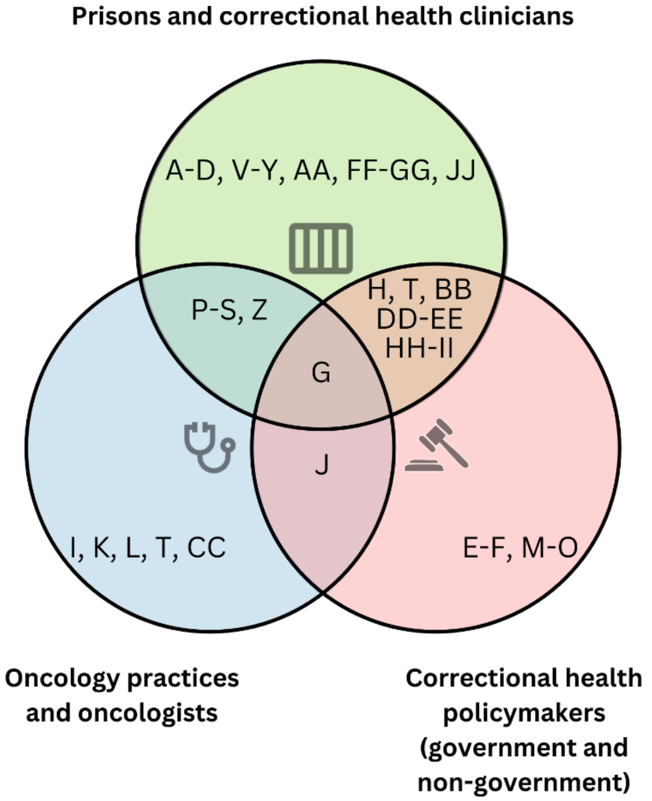

EHR = electronic health record; USPSTF = United States Preventive Services Task Force

© 2025 Manz CR et al. *JAMA Network Open*.
