# Supplementary material for: Correctional Health and Oncologist Perspectives on Strategies to Improve Cancer Care in US Prisons: A Qualitative Study
Source: JAMA Netw Open. 2025 Oct 15;8(10):e2537640. doi: 10.1001/jamanetworkopen.2025.37640 (PMC12529190; doi:10.1001/jamanetworkopen.2025.37640)
Supplement: Supplement 2. — Data Sharing Statement [file jamanetwopen-e2537640-s002.pdf]

Data Sharing Statement

Manz. Correctional Health and Oncologist Perspectives on Strategies to Improve Cancer Care in US Prisons: A Qualitative Study. *JAMA Netw Open*. Published online October 15, 2025. doi:10.1001/jamanetworkopen.2025.37640

Data
Data available: No

Additional Information
Explanation for why data not available: Individual level transcripts contain identifiable information and cannot be made available to others.
